# Serological screening for Celiac Disease in 382 pre-schoolers with Autism Spectrum Disorder

**DOI:** 10.1186/s13052-016-0308-x

**Published:** 2016-11-16

**Authors:** Sara Calderoni, Elisa Santocchi, Teresa Del Bianco, Elena Brunori, Laura Caponi, Aldo Paolicchi, Francesca Fulceri, Margherita Prosperi, Antonio Narzisi, Angela Cosenza, Raffaella Tancredi, Filippo Muratori

**Affiliations:** 1 IRCCS Stella Maris Foundation, Viale del Tirreno 331, Pisa, 56018 Calambrone Italy; 2 Stella Maris Mediterraneo Foundation, Chiaromonte, (PZ) Italy; 3 ODFlab, Department of Psychology and Cognitive Science, University of Trento, Via Matteo del Ben, 5/B, 38068 Rovereto (TN), Italy; 4 Department of Translational Research and New Technologies in Medicine and Surgery, University of Pisa, Via Savi 10, 56126 Pisa, Italy; 5 Department of Clinical and Experimental Medicine, University of Pisa, Pisa, Italy

**Keywords:** Celiac Disease, Autism Spectrum Disorders, Pre-schoolers, Screening, Autoimmune disease, Gastrointestinal symptoms, Epidemiology, Young children

## Abstract

**Background:**

Recent investigations suggest a possible common genetic background between Autism Spectrum Disorders (ASD) and Celiac Disease (CD). However, studies regarding this association are scarce and often limited by the small sample sizes and/or large heterogeneity among ASD groups in terms of demographic and clinical features. The present study aims to investigate the overall CD prevalence (biopsy proven-CD patients plus screening detected tTG and EMA positive cases) in a large population of pre-schoolers with ASD referred to a tertiary care University Hospital.

**Methods:**

We retrospectively collected data about 382 children (mean age: 46.97 ± 13.55 months; age-range: 18-72 months) consecutively diagnosed as ASD (according to the Diagnostic and Statistical Manual of Mental Disorders 4th edition criteria) over the period 2010–2013, and who performed a serological CD screening.

**Results:**

The overall CD prevalence was 2.62%, which is statistically significant higher to that reported in the Italian paediatric population (*p* = 0.0246). Half of these children had no symptoms or risk factors related to CD when they performed the serological screening.

**Conclusions:**

If replicated, these data suggest the importance of regular screening for CD in young patients with ASD, and are of relevance for clinical and public health.

## Background

Autism Spectrum Disorders (ASD) are neurodevelopmental conditions characterized by impairment in socio-communicative abilities as well as restricted and stereotyped behaviours [1]. Besides the aforementioned core symptoms, patients with ASD frequently show a wide range of associated clinical manifestations, including other psychiatric [2], and medical comorbidities [3]. Among these latter, gastrointestinal (GI) dysfunctions are reported by parents of ASD children with a rate of 20–85% depending both on definition of GI symptoms and demographic/clinical characteristics of ASD samples [4, 5]. Simultaneously, recent evidences suggest a role of immune dysfunction in ASD pathogenesis [6], sustained also by increased rates of autoimmune disorders in the families of subjects with ASD [7–9]. In this framework, a possible association between ASD and Celiac Disease (CD) has been suggested. CD is defined as “a chronic small intestinal immune-mediated enteropathy precipitated by exposure to dietary gluten in genetically predisposed individuals” [10]. The prevalence of CD in Western population is close to 1% [11]; in particular in Italy it was estimated to be 1.1% among children aged 0–16 years [12], and 1.2% among school children, on the basis of a salivary radio-immunological screening [13]. Clinical manifestations in childhood are not limited to classical GI symptoms (diarrhoea, abdominal pain, bloating, flatulence, weight loss, anorexia, constipation), but include other common or atypical manifestations potentially affecting any organ or body system. Moreover, two asymptomatic forms are described: Silent CD characterized by positive serology and histology, and Potential CD with positive serology and compatible Human Leukocyte Antigen (HLA) alleles but negative histology [14, 15]. Several complications are associated with untreated CD, and an increased risk of overall mortality has been observed [16].

The association between ASD and CD is still a matter of debate. Some studies reported no evidence for the existence of a ASD-CD link [17–19], whereas others suggested a possible connection [20], or a higher prevalence of CD in ASD children than in general pediatric population [21]. Ludvigsson and colleagues [22] found that ASD was associated with an increased risk of positive CD serology. It is worth noting that, according to one of the “gut-to-brain-connection” theories in ASD, gluten (and casein) can trigger inflammation in the gut leading to autoimmune illness or cross-reactivity with other potential central nervous system antigens [23, 24]. Therefore, the supposed relationship between gluten and ASD has paved the way for indiscriminate therapeutic approaches such as gluten (and/or casein) free diet, bereft of scientifically validated benefits in absence of a CD diagnosis, as assessed by two recent reviews [25, 26]. A further reason for the scientific community to better clarify the possible links of ASD and CD.

The aim of this study was to detect the prevalence of CD in a large series of carefully diagnosed ASD preschool children referred to a tertiary care University Hospital.

## Methods

### Participants

We retrospectively reviewed data of inpatient and day-patient preschool children who had received a first clinical evaluation with a diagnosis of Autistic Disorder, Asperger’s Disorder or Pervasive Developmental Disorder-Not Otherwise Specified according to the DSM-IV-TR criteria [27] at the IRCCS Stella Maris Foundation (Pisa, Italy) between January 2010 and December 2013. The sample was composed of 382 children, 304 males and 78 females with an average age of 46.97 months (standard deviation: 13.55 months; range: 18–72 months). ASD diagnosis was performed by a multidisciplinary team (a senior child psychiatrist, an experienced clinically trained research child psychologist, an educational therapist, and a speech-language pathologist) during 5–7 days of extensive evaluation, and confirmed by the Autism Diagnostic Observation Schedule-Generic (ADOS-G) [28] in the large majority of subjects. Exclusion criteria were: (a) neurological syndromes or focal neurological signs; (b) significant sensory impairment (e.g., blindness, deafness); (c) potential secondary causes of ASD revealed by high-resolution karyotyping, DNA analysis of Fragile-X, or screening tests for inborn errors of metabolism.

### Celiac disease screening and diagnosis

During the first evaluation in the ASD Operative Unit of our Institute, all children usually undergo a serological screening for CD as part of the routine assessment through the determination of the titres of Anti-Gliadin (AGA) immunoglobuline (Ig)A and IgG, Anti-Transglutaminase (anti-tTG) IgA, and Anti-Endomysium (EMA) IgA antibodies. The first two antibodies are quantified, respectively, with fully automated EliA™ Gliadin and EliA™ Celikey® on ImmunoCAP 250 (Phadia), and measured with fluorescent enzyme immunoassay (FEIA). The upper limit of their physiological range is 10 U/ml values; between 7 and 10 U/ml are considered “borderline”; values under 7 U/ml are considered negative. EMA IgA is measured with indirect immunofluorescence on Euroimmun slides, and it is subject to significant variability of interpretation, but its specificity is very high (98–100%) [15]. Possible EMA results in our sample were: “+”: positive result; “-”: negative result; “+/-”: doubt/borderline result. We retrieved the results of the CD screenings from the digital database of the Clinical Pathology Laboratory of St Chiara Hospital (Pisa, Italy). According to the European Society for Pediatric Gastroenterology, Hepatology, and Nutrition (ESPGHAN) new guidelines for the CD diagnosis [15, 29], the serological diagnosis of CD is based on the detection of class IgA anti-tTG and EMA antibodies; in patients with IgA deficiency, IgG anti tTG is used. Specifically, the sensitivities for both tTG and EMA antibodies ranged from 70 to 100% [30]. In a large, prospective, biopsy-confirmed study on adult patients without a previous known diagnosis of CD, the negative predictive value for IgA tTG was 99.6%, and it increased to 99.7% when a two-step approach was adopted, using tTG first and EMA then [31]. The specificity for tTG ranged from 91 to 100%, whereas EMA specificity is even higher (98–100%), so much that this test is considered specific for a diagnosis of CD [30]. A positivity of both anti-tTG and EMA antibodies has a sensitivity and positive predictive value for CD close to 100% [30, 32]. A confirmation of the CD diagnosis is recommended to all individuals positive for anti-tTG and/or EMA antibodies. Therefore, we selected from the sample all subjects with positive or borderline values of anti-tTG and/or EMA antibodies at the serological screening and then checked in the patient’s medical records or through call interviews whether the CD diagnosis had been subsequently confirmed by paediatric gastroenterologists. We did not took into account the screening levels of AGA IgA and IgG antibodies for the purposes of this study since they have been recently considered as the less specific markers of inflammation due to dietary gluten, their increase may be explained by an array of diseases and its significance is still under debate [15, 29].

Since we aimed to compare the overall prevalence of CD in our sample to that found in the general population, we used the data from the survey of Mustalahti et al. [12], which investigated the CD prevalence in four European countries -Italy included- by analyzing the sera from 29,212 children and adults. The Italian sample of children was composed of 2649 individuals with an age-range from 0 to 19 years (the means and standard deviations values were not reported). The age range of our sample was quite different from that of this European survey [12] (1.5–6 years versus 0–19 years). Therefore, since the prevalence of CD tends to increase by age [33], an underestimation of CD prevalence in our ASD population could not be excluded when it is compared to the prevalence of the Italian children included in the European survey, i.e. 1.1% (95% CI: 0.7–1.5), derived from “previously diagnosed biopsy-proven CD patients plus screening detected individuals with both anti-tTG and EMA positivity” [12].

### Statistical analysis

Statistical calculations were performed with SPSS® version 19.0. We performed the Chi-square test to compare the prevalence of CD between our sample and the Italian pediatric population [12]. A *p*-value <0.05 was considered significant.

## Results

Among 382 pre-schoolers with ASD, the retrospective review of their medical records identified ten patients with CD or with positive CD serology (Table 1). In particular, nine subjects resulted serologically positive for anti-tTG IgA and/or EMA antibodies during their first neuropsychiatric evaluation. In seven of these children (patients 1–7), the diagnosis of CD was confirmed by paediatric gastroenterologists: (a) through multiple duodenal biopsies (subjects 1–5); (b) on the basis of very high anti-tTG antibody levels and HLA positivity (patient 6); (c) on the presence of Duhring-Brocq dermatitis herpetiformis, a chronic bullous disease specifically correlated with sensitivity to gluten enteropathy and CD (patient 7) [34]. All these patients were on a gluten-free diet (GFD). As far as patient 8 is concerned, we were not able to obtain any further information since his parents were no more contactable after the serological screening. The patient 9 did not perform further evaluations even in the presence of repeated positivity of CD serology due to parents’ refusal. The patient 10 was already diagnosed as celiac and was on a GFD at the time of the first clinical evaluation in our hospital. She had performed CD serological screening at the age of 32 months since she showed growth failure and dental enamel defects. She resulted serologically positive for anti-tTG IgA and EMA antibodies, and eventually CD diagnosis was confirmed by multiple duodenal biopsies and HLA positivity.

**Table 1:** Serology, clinical data and diagnosis confirmation of the ten ASD subjects with CD or positive CD serology

No.	Age (months)	Sex	tTG Ig A (U/ml)	EMA	AGA IgA (U/ml)	AGA IgG (U/ml)	Risk factor and clinical presentation	CD diagnosis
1	27	F	>200.0	+	15.0	127.0	Inappetence	MDB +
2	67	M	40.7	+	8.0	18.9	None	MDB +
3	41	M	35.0	+	9.3	41.0	CD in the sister	MDB +
4	44	M	102.0	+	1.4	1.1	None	MDB +
5	24	F	11.6	-	0.2	6.4	Diarrhea	MDB +
6	60	M	>200	+	93.0	85.5	None	HLA +
7	44	F	3.6	+/-	1.2	17.0	Growth failure	DH
8	20	M	23.0	+	1.4	9.0	None	n.p.
9	44	M	15.0	+	1.6	9.6	None	n.p.
10^a^	58	F	n.a.	n.a.	n.a.	n.a.	Growth failure Dental enamel defects	MDB +, HLA +

*M* male, *F* female, *tTG Ig A* anti-transglutaminase antibodies, *EMA* anti-endomysium antibodies, *AGA IgA and IgG*: anti-gliadin antibodies; cut -off values for tTG IgA, AGA IgA and AGA IgG: positive: > 10U/ml; borderline: 7–10 U/ml, negative: <7 U/ml; EMA: “+”: positive result; “-“: negative result; “+/-“: doubt result; *n.p.* not performed, *n.a.* not available, *HLA+* human leukocyte antigen positivity, *MDB +* multiple duodenal biopsies positivitym, *DH* Duhring Dermatitis Herpetiformis

^a^ASD patient who had received a CD diagnosis before the hospitalization in our ASD Unit and for which serological data are not available

All in all, patients with CD or with positive CD serology were ten, six males and four females, with mean age of 41 months (SD: 15.2; range: 20–67). Therefore, the prevalence of “CD patients *plus* screening detected individuals with both anti-tTG and EMA positivity” was 2.62% (10/382 subjects).

The Chi square test (see Table 2) indicated a statistically significant difference (*p* value = 0.0131) in the prevalence of CD cases identified in our sample (2.62%; 95% CI: 1.0–4.2) as compared to the sample obtained from the Italian paediatric population (1.1%; 95% CI: 0.7–1.5) [12].

**Table 2:** Comparison between the prevalence of celiac disease (CD) in our sample and in the Italian pediatric population (Mustalahti et al., [12])

	Outcome 1# Number of subjects	Outcome 2# Number of subjects	Total Number of subjects	Chi-square statistic value
Group 1 Our study	10	372	382	0.0131*
Group 2 Mustalahti et al., 2010 [12]	29	2616	2645	
Total	39	2988	3027	

*The result is significant at *p* <0.05

1# Children with celiac disease (CD) defined as “previously diagnosed biopsy-proven CD patients plus screening detected individuals with both anti-tTG and EMA positivity”

2# Children without CD defined as “previously diagnosed biopsy-proven CD patients plus screening detected individuals with both anti-tTG and EMA positivity”

Of the ten subjects with CD (or positive CD serology), four patients (40%: the patients No 1, 5, 7, and 10) showed one or more symptoms suggestive of a typical form of CD. Patient No 3 was asymptomatic, but belonged to a high risk group given a positive familiarity for CD. Five patients (50%) had no symptoms or risk factors correlated with CD at the time of the serological screening.

## Discussion

Our results indicate that an association between ASD and CD cannot be excluded. The comparison of our findings with those of previous studies is complex given several differences in the study design (prevalence of ASD in CD children or *viceversa*), in CD serological screening methodologies (only tTG, both EMA and tTG, AGA and/or EMA and/or tTG antibodies positivity), and in the ASD samples (in terms of size, age of patients, severity of ASD symptoms, diagnostic evaluation and diagnostic criteria). The first studies were performed in the 70’ies [35, 36] and they both did not find any significant association between autism and CD. Some more recent studies also rejected the ASD-CD link. In particular, Batista et al. [17] found no significant differences in the prevalence of CD in 147 ASD patients compared to a group of 2034 children and adolescents originating from the same geographical region and from a similar low-income stratum. In the same work, the prevalence of ASD in 211 patients with CD was 0.95% and therefore not significantly different from the prevalence of 0.9% found in the general US population. Pavone et al. [18], in a case-control study, found no cases of autism in 120 patients with CD, and no cases of CD in 11 patients with autism. On the contrary, Barcia et al. [21] studied retrospectively a large population of 150 randomly selected patients with ASD, and found an increased prevalence of intestinal biopsy-confirmed CD in ASD children (3.3%) compared to the normal population (0.9%). This prevalence (3.3%) is quite similar to that found in our work (2.62%). It is to note that, while Barcia and colleagues included only biopsy-confirmed CD patients, we considered as CD patients also the two children without a histological confirmation of the diagnosis, but with both tTG and EMA positive antibodies. We made this choice in agreement with some authors [12] who recently argued that “even if the small intestinal biopsy has an indisputable diagnostic role in the clinical setting, this invasive investigation is not an essential requirement for an epidemiological survey”. Besides, in several epidemiological studies the antibody positivity has been considered as the only criterion for CD diagnosis [37–39]. In another recent work, Ludvigsson et al. [22] collected data on 26,995 individuals with CD, 12,304 individuals with inflammation, and 3719 individuals with normal mucosa but positive CD serology (AGA IgA/IgG, EMA and tTG), and compared them with 213,208 age- and sex-matched controls. They found that having a prior diagnosis of ASD was not associated with CD (OR = 0.93) or inflammation (OR = 1.03), but was related with a markedly increased risk of positive CD serology (OR = 4.57) [22]. Our findings differed from Ludviggson and colleagues [22] as for the possible association of ASD and CD but it is to note that they estimated the prevalence of ASD diagnosis in subjects that had just performed an intestinal biopsy, while we examined the prevalence of CD diagnosis after a serological screening in a cohort of subjects diagnosed as ASD. Moreover, the definition of positive CD serology also differed between Ludviggson et al. [22] and the current investigation since they included subjects with positive AGA IgG and IgA antibodies, while we did not take into account the values of these antibodies. In fact, the main purpose of the present study was to define the prevalence of CD, and AGA antibodies are now considered as the less specific markers of CD in the serological screenings [15].

Among the GI symptoms reported by parents of children with ASD and CD in our sample there were diarrhea and inappetence. The former is one of the most frequently reported GI symptoms in ASD children in several studies [5, 40] while the latter has been recently detected as one of the most common GI symptoms in a large sample of pre-schoolers with ASD [41]. Besides, half of the ASD children with CD were asymptomatic at the time of the serological screening: this prevalence could be ascribed not only to the presence of true asymptomatic forms of CD but also to the severe communication difficulties of non-verbal preschool children with ASD, making them unable to express GI and systemic symptoms suggestive of CD (e.g. recurrent abdominal pain, abdominal distension, chronic fatigue) [4, 42]. Otherwise, pre-schoolers with ASD could show their GI-related distress in alternative ways through a greater severity of problem behaviors such as irritability [43], anxiety and affective problems [44, 45], or externalizing behaviors (oppositional defiant behaviors and tantrums) [46]. These clinical data contributed to highlight the importance of a serological screening for CD in young children with ASD, even in absence of clear GI or systemic symptoms or other risk factors related to CD.

A possible limitation of the current study was the absence of data about total IgA level on all ASD patients. Consequently, a certain number of children with CD and anti IgA deficiency could be not detected leading to an underestimation of CD prevalence in our ASD population. Another possible source of CD prevalence underestimation in our ASD sample could be ascribed to the young age of patients included that does not allow to identify ASD individuals at genetic risk for CD who develop CD only later. Moreover, we were not able to provide ADOS-G [28] scores, since this instrument was originally used for clinical purposes only (in order to confirm the ASD diagnosis), and therefore his scores were not usable for research purposes.

## Conclusion

Our results indicate that children with ASD may be at increased risk of CD. This finding has implications beyond medical status for children with ASD. Specifically, the fact that GI symptoms suggestive of CD are difficult to express in words by non-verbal or minimally-verbal preschoolers with ASD implies a possible manifestation of GI-related distress through behavioral symptoms, including increased anxiety, decreased social responsiveness, temper tantrums, opposition, sleep problems, food selectivity, aggressive and self-injurious acts [4, 41, 47, 48]. In these cases, if a diagnosis of CD is confirmed, the treatment of ASD patients for CD with a gluten-free diet may not only alleviate their CD-related symptoms, but also have a positive impact on associated behavioral problems. Therefore, the screening for CD in young patients with ASD could be important for decreasing current and long-term morbidity, as well as increasing overall health and quality of life.

## Abbreviations

AGA: Anti-gliadin
ASD: Autism spectrum disorders
CD: Celiac disease
EMA: Anti-endomysium
HLA: Human leukocyte antigen
Ig: Immunoglobuline
tTG: Transglutaminase
